# Microbiome-driven IBS metabotypes influence response to the low FODMAP diet: insights from the faecal volatome

**DOI:** 10.1016/j.ebiom.2024.105282

**Published:** 2024-08-22

**Authors:** Thomas Edward Conley, Rachael Slater, Stephen Moss, David Colin Bulmer, Juan de la Revilla Negro, Umer Zeeshan Ijaz, David Mark Pritchard, Miles Parkes, Chris Probert

**Affiliations:** a University of Liverpool Institute of Systems, Molecular and Integrative Biology, Liverpool, UK; b Liverpool University Hospitals NHS Foundation Trust, Department of Gastroenterology, Liverpool, UK; c Cambridge University Hospitals NHS Foundation Trust, Department of Gastroenterology, Cambridge, UK; d University of Cambridge Department of Medicine, Gastroenterology and Hepatology, Cambridge, Cambridgeshire, UK; e University of Cambridge Department of Pharmacology, Cambridge, Cambridgeshire, UK; f University of Glasgow, Mazumdar-Shaw Advanced Research Centre, Glasgow, UK

**Keywords:** Irritable bowel syndrome, Low FODMAP diet, Metabolome, Metabotype, Microbiome, Short chain fatty acids, Volatile organic compounds

## Abstract

**Background:**

Irritable bowel syndrome (IBS) is a common and debilitating disorder manifesting with abdominal pain and bowel dysfunction. A mainstay of treatment is dietary modification, including restriction of FODMAPs (fermentable oligosaccharides, disaccharides, monosaccharides and polyols). A greater response to a low FODMAP diet has been reported in those with a distinct IBS microbiome termed IBS-P. We investigated whether this is linked to specific changes in the metabolome in IBS-P.

**Methods:**

Solid phase microextraction gas chromatography-mass spectrometry was used to examine the faecal headspace of 56 IBS cases (each paired with a non-IBS household control) at baseline, and after four-weeks of a low FODMAP diet (39 pairs). 50% cases had the IBS-P microbial subtype, while the others had a microbiome that more resembled healthy controls (termed IBS-H). Clinical response to restriction of FODMAPs was measured with the IBS-symptom severity scale, from which a pain sub score was calculated.

**Findings:**

Two distinct metabotypes were identified and mapped onto the microbial subtypes. IBS-P was characterised by a fermentative metabolic profile rich in short chain fatty acids (SCFAs). After FODMAP restriction significant reductions in SCFAs were observed in IBS-P. SCFA levels did not change significantly in the IBS-H group. The magnitude of pain and overall symptom improvement were significantly greater in IBS-P compared to IBS-H (*p* = 0.016 and *p* = 0.026, respectively). Using just five metabolites, a biomarker model could predict microbial subtype with accuracy (AUROC 0.797, sensitivity 78.6% (95% CI: 0.78–0.94), specificity 71.4% (95% CI: 0.55–0.88).

**Interpretation:**

A metabotype high in SCFAs can be manipulated by restricting fermentable carbohydrate, and is associated with an enhanced clinical response to this dietary restriction. This implies that SCFAs harbour pro-nociceptive potential when produced in a specific IBS niche. By ascertaining metabotype, microbial subtype can be predicted with accuracy. This could allow targeted FODMAP restriction in those seemingly primed to respond best.

**Funding:**

This research was co-funded by 10.13039/501100002927Addenbrooke's Charitable Trust, Cambridge University Hospitals and the Wellcome Sanger Institute, and supported by the 10.13039/501100018956NIHR Cambridge Biomedical Research Centre (BRC-1215-20014).

**Research in context**

**Evidence before this study**

Irritable bowel syndrome (IBS) is estimated to affect at least 4% of the general population. The cardinal symptoms of IBS are abdominal pain related to defaecation, and associated changes in stool form and/or frequency. Dietary intervention is encouraged early in the algorithmic approach to IBS, and the low FODMAP (fermentable oligosaccharides, monosaccharides, disaccharides and polyols) diet, though relatively resource-intensive and challenging to follow, is considered the most effective dietary strategy. Whilst most patients demonstrate a clinical response, some respond better than others; yet, the ability to identify higher magnitude responders at baseline remains elusive. A biomarker (or bioprofile) to identify higher-magnitude responders could help justify the challenges/resource-burden of this approach, directing dietetic resource toward those likely to benefit most. A key driver underpinning the efficacy of the low FODMAP diet is the consequent alteration in microbiota derived metabolites. Indeed, recent evidence demonstrates that the magnitude of response to FODMAP restriction varies significantly depending on microbiota composition, with those enriched in microbes primed to ferment carbohydrate responding best. Further, a distinct metabotype characterised by a relative abundance of short chain fatty acids (SCFAs) is emerging in the literature, again associating with enhanced response to FODMAP restriction. How this metabotype relates to microbial composition remains unclear.

**Added value of this study**

In this study we used solid-phase microextraction gas-chromatography mass-spectrometry to analyse the volatile organic compounds (VOCs) emitted in the faecal headspace of IBS patients and their healthy non-IBS household controls. Patients had already been categorised into two distinct microbial subgroups, each responding in different orders of magnitude to FODMAP restriction. Two distinct metabotypes emerged, mapping onto each microbial subtype and correlating strongly with bacterial metabolic pathways. A metabotype enriched in SCFAs at baseline characterised the microbial subtype within which an enhanced clinical response to FODMAP restriction was observed. Following FODMAP restriction in this group, the SCFA abundance was normalised to levels seen in healthy non-IBS controls. Using just five of the most influential VOCs characterising the two metabotypes, a biomarker model was able to predict microbial subtype membership with accuracy. Taken together, the results of the present study illustrate the relationship between the gut microbiome and the metabolome in IBS, and provide valuable insights into the biological mechanisms underpinning the spectrum of clinical response observed with FODMAP restriction.

**Implications of all the available evidence**

The low FODMAP diet is a well-known, commonly used intervention in the management of IBS, however concerns have arisen regarding its potential to deplete the gut of key SCFA metabolites. The observed dynamics of SCFA in this study provide reassurance that, although abundance does drop, the observed ‘drop’ is a regression to normal. The analysis of faecal VOCs offers a cost-effective means of examining the functional gut microenvironment, and the present study demonstrates an ability to infer microbial subtype and function using metabolomic analysis. The observed ability to predict microbial subtype using a metabolic biomarker model is a promising step toward much-needed personalised IBS care. Larger confirmatory studies with high discriminatory power are now warranted. Future studies should focus on the potentially pathophysiological role of SCFA in IBS, and the long-term impacts of FODMAP restriction on these key gut metabolites.

## Introduction

Irritable bowel syndrome (IBS) affects at least 4.1% of the population globally and in the United Kingdom (UK) annual direct healthcare costs are estimated to exceed £1.3 billion.^1^^,^^2^ Moreover, IBS substantially impairs quality of life.^3^ Although various treatment strategies exist for the management of IBS,^4^ most are empirical and the pre-treatment identification of ‘therapy-responders’ remains elusive. This is particularly pertinent when considering the low FODMAP (fermentable oligosaccharides, monosaccharides, disaccharides and polyols) diet—which is effective in reducing symptom burden,^5^ but challenging to follow and can be expensive.^6^

Understanding “what changes in diet benefit people with IBS” and whether “treatments which balance the gut bacteria are effective for people with IBS” have recently been identified as two of the top-ten research priorities in IBS care.^7^ Amongst other mechanisms that underpin the association between the consumption of fermentable carbohydrates and symptom expression in IBS,^8^ the interaction between dietary FODMAPs and the gut microbiome is increasingly recognized.^9^, ^10^, ^11^, ^12^ Furthermore, the manipulation of the microbiome influences both symptom profile and severity in IBS, albeit to varying degrees.^12^^,^^13^ However, an IBS-specific, reproducible microbial signature is yet to emerge.

Though gut microbiota composition is subject to increasing attention in the IBS literature, it is through consequent changes in the abundance and amalgam of the microbiota-derived metabolites (termed the gut metabolome) that physical symptoms may become manifest. Indeed, treatments targeting key pro-nociceptive neuro-active metabolites such as histamine^10^^,^^14^^,^^15^ and serotonin^16^^,^^17^ have proven beneficial in IBS. Recently, the short chain fatty acids (SCFAs, acetic, propanoic, butanoic and pentanoic acid) and branched-SCFAs (2-methylpropanoic, 2-methylbutanoic and 3-methylbutanoic acid), mainly produced from the microbial fermentation of indigestible carbohydrates and amino acid metabolism, have been implicated as having pro-nociceptive potential when produced in an IBS ecosystem.^18^^,^^19^ The metabolomic analysis of faecal volatile organic compounds (VOCs–including key metabolites such as SCFAs) offers a cost-effective means of examining the functional gut microenvironment.

Vervier et al. (2022) recently demonstrated the presence of two distinct gut microbial subtypes in a cohort of patients with diarrhoea-predominant or mixed-type IBS. The microbiome of one group was more closely associated with that observed in non-IBS controls (termed IBS-H, ‘*healthy’*).^12^ The other demonstrated a less diverse, relatively ‘dysbiotic’ microbiome (termed IBS-P, ‘*pathological’*). The microbiota in this group was depleted in commensal species from the Bacteroidetes phylum and enriched in species from the Firmicutes phylum, harbouring pathways for amino acid (including tryptophan and histidine) biosynthesis and carbohydrate metabolism/SCFA biosynthesis. Those individuals with the IBS-P subtype not only demonstrated higher symptom/pain severity scores at baseline, but also showed a more marked decrease in scores following FODMAP exclusion. Alongside these clinical findings, a dynamic shift in the IBS-P microbiota composition occurred after FODMAP exclusion; functional profiling using pathway enrichment suggested that metabolites might be impacted as a consequence of this shift.

We now expand the work of Vervier et al. providing VOC metabolomic analysis with the aim of gaining further insight into whether these two microbial subtypes correspond with two distinct and measurable metabotypes. It is hypothesised that the components of, and dynamic changes within, these (and indeed other) metabotypes will further support the notion that specific microbial subtypes harbour a ‘pro-IBS’, and potentially a pro-nociceptive, bowel environment in this group of subjects.

## Methods

The current analysis used clinical data and stool samples collected and curated by Vervier et al. (2022).^12^ What follows is a summary of that study design. Due to the sequential nature of our analysis, slight adjustments to the initial study design were unavoidable (for example—depletion of faecal matter available for analysis).

### Study participants

Study participants were recruited into a single centre (Cambridge University Hospital, UK) case–control study between 2016 and 2019. Gender was reported according to biological sex status. Cases met Rome IV criteria for IBS diarrhoeal and mixed sub-classes. Individuals with IBS-constipation were excluded from recruitment during the index study to avoid introducing an obvious transit-related microbial batch-effect into the analysis. All cases were paired with a healthy (non-IBS) household control. Baseline characteristics are described in Table 1. Inclusion and exclusion criteria are summarised in Table 2.

**Table 1:** Demographics of study participants with IBS.

	Cases vs healthy household controls			Case comparisons: IBS-P vs IBS-H		
	Cases	Controls	*p* value	IBS-P	IBS-H	*p* value
Case number	56	56	–	28	28	–
Age (mean ± SD)	39.3 (±13.4)	44.7 (±14.8)	<0.05^a^	37.4 (±12.5)	39.9 (±14.4)	ns^a^
Age distribution (%)						
<40	32 (57.1)	22 (39.3)	ns^b^	16 (57.1)	16 (57.1)	ns^b^
40–59	20 (35.7)	25 (44.6)	ns^b^	10 (35.8)	10 (35.8)	ns^b^
>60	4 (7.1)	9 (16.1)	ns^b^	2 (7.1)	2 (7.1)	ns^b^
Female (%)	41 (73.2)	19 (33.9)	<0.01^a^	22 (78.6)	19 (67.9)	ns^b^
Male (%)	15 (26.8)	37 (66.1)		6 (21.4)	9 (32.1)	
BMI (mean ± SD)	27.9 (±6.8)	NR	–	29.4 (±7.7)	26.5 (±5.4)	ns^a^
BMI distribution (%)						
<18.5	1 (1.8)	N/A	–	1 (3.6)	0 (0.0)	N/A
18.5–24.9	21 (37.5)	N/A	–	6 (21.4)	15 (53.6)	<0.05^b^
25.0–35.0	27 (48.2)	N/A	–	16 (57.1)	11 (39.3)	ns^b^
>35.0	7 (12.5)	N/A	–	5 (17.9)	2 (7.1)	ns^b^
Smokers (%)	5 (8.9)	0 (0.0)	ns^a^	4 (14.3)	1 (3.6)	ns^a^
Median weekly alcohol intake in units (range)	3.0 (0.0–28.0)	5.0 (0.0–31.0)	ns^b^	3.5 (0.0–28.0)	0.5 (0.0–20.0)	ns^c^
Median FODMAP score (range)	8.0 (3.0–13.0)	8.0 (0.0–13.0)	ns^b^	8.0 (5.0–12.0)	8.0 (3.0–13.0)	ns^c^

	Case comparisons: IBS-P vs IBS-H		
	IBS-P	IBS-P	IBS-P
IBS subtype (%)			
IBS-Diarrhoea	12 (42.8)	11 (39.3)	ns^b^
IBS-Mixed	16 (57.2)	17 (60.7)	ns^b^
Post-infectious IBS (%)	8 (28.6)	8 (28.6)	ns^b^
Baseline IBS-SSS (mean ± SD)	300 (±83.1)	261 (±86.1)	ns^a^
IBS-SSS distribution (%)			
<75	0 (0.0)	0 (0.0)	–
75–174	2 (8.0)	3 (11.1)	–
175–299	10 (40.0)	16 (59.3)	–
300–500	13 (52.0)	8 (29.6)	–
Baseline IBS-SSS pain composite score (--/200) (mean ± SD)	117 (±50.2)	91 (±49.1)	ns^a^

Sex defined by biological female/male status. (BMI, body mass index; FODMAP, fermentable oligosaccharides, disaccharides, monosaccharides and polyols; IBS, irritable bowel syndrome; IBS-H, ‘healthy’ microbial subtype; IBS-P, ‘pathological’ microbial subtype; IBS-SSS, irritable bowel syndrome severity scoring system; N/A, not applicable; NR, not recorded; ns, non significant; SD, standard deviation).

a Independent samples t test.

b Chi squared test.

c Mann Whitney U test.

**Table 2:** Study inclsuion/exclusion criteria.

Inclusion criteria	Exclusion criteria
Age 18–68 years	Pregnancy
Rome IV IBS-D or IBS-M	Restrictive diets (Low FODMAP, gluten free etc)
Cohabitation with healthy household control	Probiotic use
	Use of drugs capable of manipulating the microbiome in previous four-weeks: ▪ Antibiotics ▪ Proton pump inhibitors ▪ Colonoscopy preparation ▪ Metformin

### Study design

**Fig. 1:**
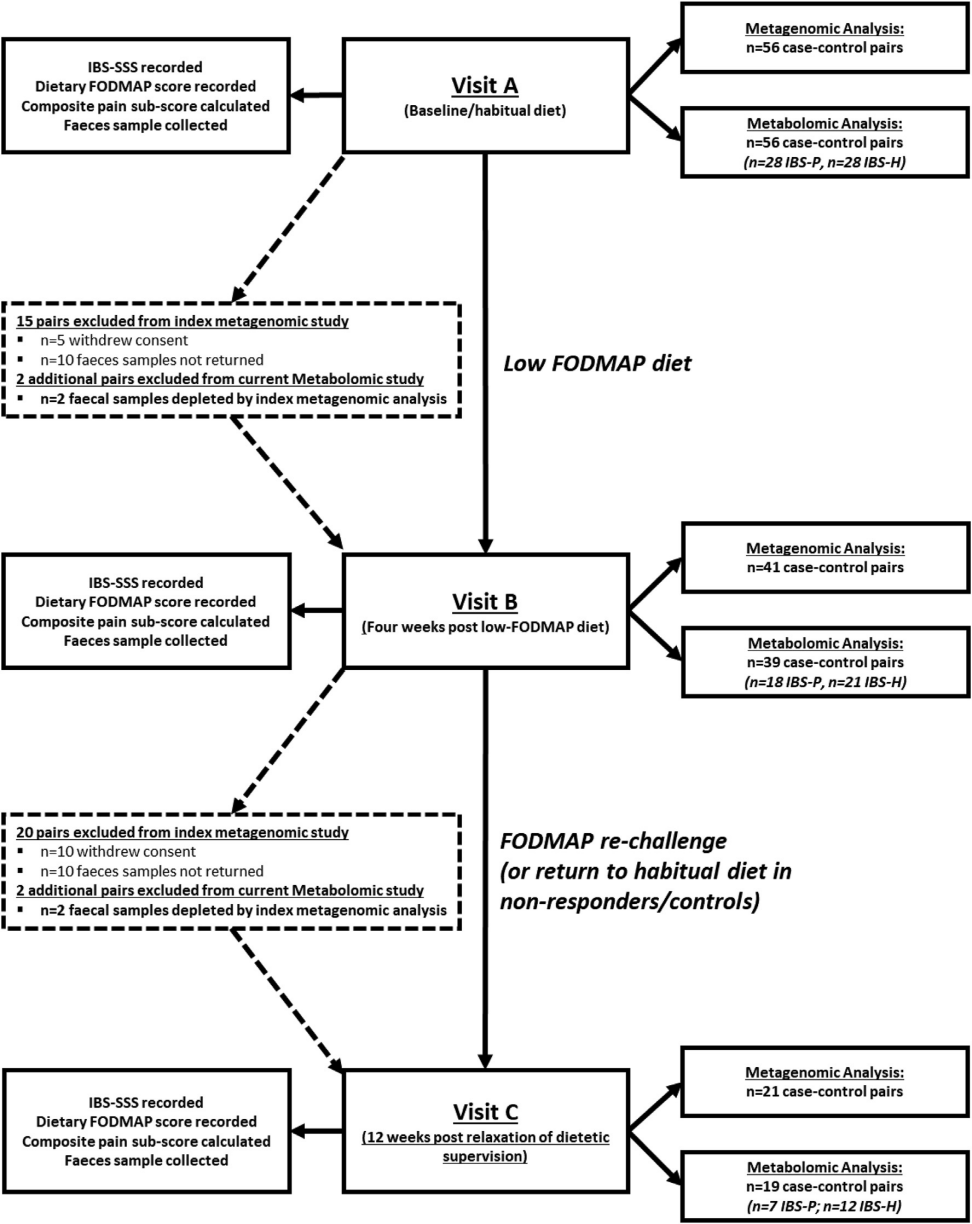
Flow chart outlining study design. At each time point, the samples available for analysis are reported for both the index study conducted by Vervier et al. (2022) and the current metabolomic study. The slight increase in drop out in the metabolomic study reflects the inability to perform metabolomic analysis due to biological sample depletion (FODMAP, fermentable oligo-, di-, mono-saccharides and polyols; IBS-SSS, IBS symptoms severity scale).

Study design follows that published by Vervier et al. and is summarised in Fig. 1. Baseline FODMAP consumption and the degree of FODMAP exclusion on the low FODMAP diet was calculated after the appraisal of seven-day food diaries.^9^ Symptom severity and response were assessed using the IBS-severity scoring system (IBS-SSS)^20^ (Supplementary Material 1), from which a composite score assessing pain was calculated.

### Sample processing

Samples were stored at −80 °C until analysis. Frozen stool was aliquoted into 10 ml headspace vials with silicone septa (Supelco, Germany).

### Faecal headspace volatile organic compound analysis

All samples were analysed by headspace solid-phase microextraction gas-chromatography mass-spectrometry (GC–MS) using a PerkinElmer Clarus 500 GC–MS quadrupole benchtop system (PerkinElmer, Beaconsfield, UK) and a Combi PAL auto-sampler (CTC Analytics, Switzerland). The GC column was Zebron ZB-624, inner diameter −.25 mm, length 60 m, film thickness 1.4 μm (Phenomenex, Macclesfield, UK), and the fibre used was Divinylbenzene/Carboxen/Polydimethylsiloxane (DVB/CAR/PDMS) 50/30 μm (1 cm) (Sigma–Aldrich, Dorset, UK).

An optimized method published elsewhere by Reade et al. (2014)^21^ was utilized throughout (Supplementary Material 2). Samples were run in a randomised order maintaining household-pairing (Supplementary Material 3).

### Data processing

All GC–MS data were processed using a pipeline system involving: i) The Automated Mass Spectral Deconvolution and Identification System software (AMDIS, Version 2.73, 2017)—used to deconvolute chromatograms and identify potential VOCs. Analysis settings within AMDIS were optimised for faecal VOC analysis in accordance with previous comparable work published elsewhere.^22^ ii) The NIST Mass Spectral Search Program and Library (Version 2.3, 2017)–used to assign VOCs. iii) The R (Version 4.2.2, 2022) package Metab–used to further align the metabolites identified according to the retention times, and to determine their relative abundance based on ionic intensities.^23^

The combination of the AMDIS and NIST software enabled the curation of an in-house VOC library. Assignment of compounds were made using NIST (Supplementary Material 4). Compounds were named according to IUPAC (International Union of Pure and Applied Chemistry) nomenclature.

### Statistical analysis

All statistical analyses were performed using ‘R’ (Version 4.2.2, 2022) and IBM® SPSS® Statistics 24 (Version 28.0.1.1).

The primary analyses focused on assessing VOC abundance profiles and the dynamic changes in VOC levels after FODMAP restriction. Secondary analyses explored VOC correlation patterns and the development of predictive models for baseline class classification. In these correlation analyses, we examined both ‘VOC-VOC’ relationships and ‘VOC-bacterial metabolic pathway’ interactions.

Where raw data were categorical (such as the binary metric of ‘absolute VOC presence/absence’), Chi Squared test and McNemar's test were used to identify preliminary differences groups prior to pre-processing. For analysis of VOC abundance—data were subject to pre-processing. First, the dataset was filtered to ensure statistical models were robust; variables were excluded from the dataset when the proportion of missing elements accounted for more than 50% among each biological group. Missing data values were replaced by imputation with 20% of the lower limit of detection in the dataset for each VOC concerned. Second, relative abundance data were normalised according to their median value, log-transformed (base 10) and auto-scaled (mean-centred and divided by the standard deviation of each variable). Data analysis was then undertaken using the online platform, MetaboAnalyst 5.0 (Canada, Version 12.0).

The distribution of VOC abundance data was assessed by Shapiro Wilk test and visual inspection of histograms/Q–Q plots). Univariate analysis used Wilcoxon rank sum/Wilcoxon signed-rank tests and Kruskall–Wallis tests where data followed a non-parametric distribution, otherwise T-tests (paired and independent) were used. When moving from one study point to another, significant changes in VOC abundance were determined using paired univariate analysis, and the magnitude of these changes were determined by calculating log_2_ fold-change. Significantly altered metabolites were initially defined by an unadjusted *p* value < 0.05. False discovery rates (FDR) were then calculated to reduce the risk of type I error and are reported as ‘*q* values’.

Heatmaps were generated to visually compare VOC profiles between groups, and where practical, receiver operating characteristics (ROC) were assessed. Integrated analysis to combine baseline VOC and metagenomic MetaCyc pathway data (reported previously, Vervier et al., 2022) was performed using the Data Integration Analysis for Biomarker discovery (DIABLO) framework in the R mixOmics package to provide visual representation of the relationships between VOCs and the bacterial metabolic pathways.^24^ Specific parameters are described in Supplementary Material 5.

### Ethics

Approval was provided by Cambridge Central Research Ethics Committee reference 15/LO/2128. All study participants provided informed written consent.

### Role of funders

Support for the collection of biological samples was provided by Addenbrooke's Charitable Trust and support with the index metagenomic analysis was provided by the Wellcome Sanger Institute. Funders played no role in study design, data collection, data analyses, interpretation, or writing of report.

## Results

### Cohort summary

112 participants (56 cases [IBS-P, n = 28; IBS-H, n = 28], 56 controls) were included in the study. Similar proportions of IBS-D and IBS-M were distributed throughout each microbial subgroup (Table 2). Baseline FODMAP consumption was similar in cases and controls, and after the four-week low FODMAP diet, FODMAP scores were substantially reduced (Cases—median FODMAP score 0, IQR 0–1; Controls—median FODMAP score 0, IQR 0–1).

### Symptom severity, pain and responsiveness

More patients with IBS-P had severe IBS symptoms (Supplementary Material 6). More individuals with IBS-P than IBS-H achieved remission following the low FODMAP diet (43.8% vs 25.0%; OR 2.30, 95% CI [1.26, 4.21], *p* < 0.005 [Chi squared test]). There was no significant difference in the response rates between the two IBS microbial subtypes using the 50-point IBS-SSS delta, but the magnitude of response (absolute [and percentage] IBS-SSS delta) was greater in patients with the IBS-P subtype than the IBS-H subtype (mean delta 193.8 [56.9%] vs 119.6 [39.6%], respectively; *p* < 0.05 [independent samples t-test; Cohen's d 0.722]).

Although mean baseline pain scores were higher in cases with IBS-P (117/200) than in those with IBS-H (91/200), the difference was not significant (*p* = 0.065). In both groups, there was a significant reduction in the pain score after low-FODMAP dietary intervention. This change was greater in those with IBS-P compared with IBS-H (mean delta 79.5 [63.1%] vs 42.1 [38.1%], respectively; *p* < 0.05 [independent samples t-test; Cohen's d 0.689]).

When comparing individuals according to their Rome criteria subtypes, IBS-D vs IBS-M–there were no significant differences (independent samples t-test) in mean baseline symptom severity (286.5 [SD ± 80.8] vs 265.3 [SD ± 86.3]), response according to either the 50-point IBS-SSS delta (85.7% vs 76.7%) or the magnitude of IBS-SSS delta (151.3 [SD ± 80.0] vs 145.7 [SD ± 106.9]).

### Baseline diet

**Fig. 2:**
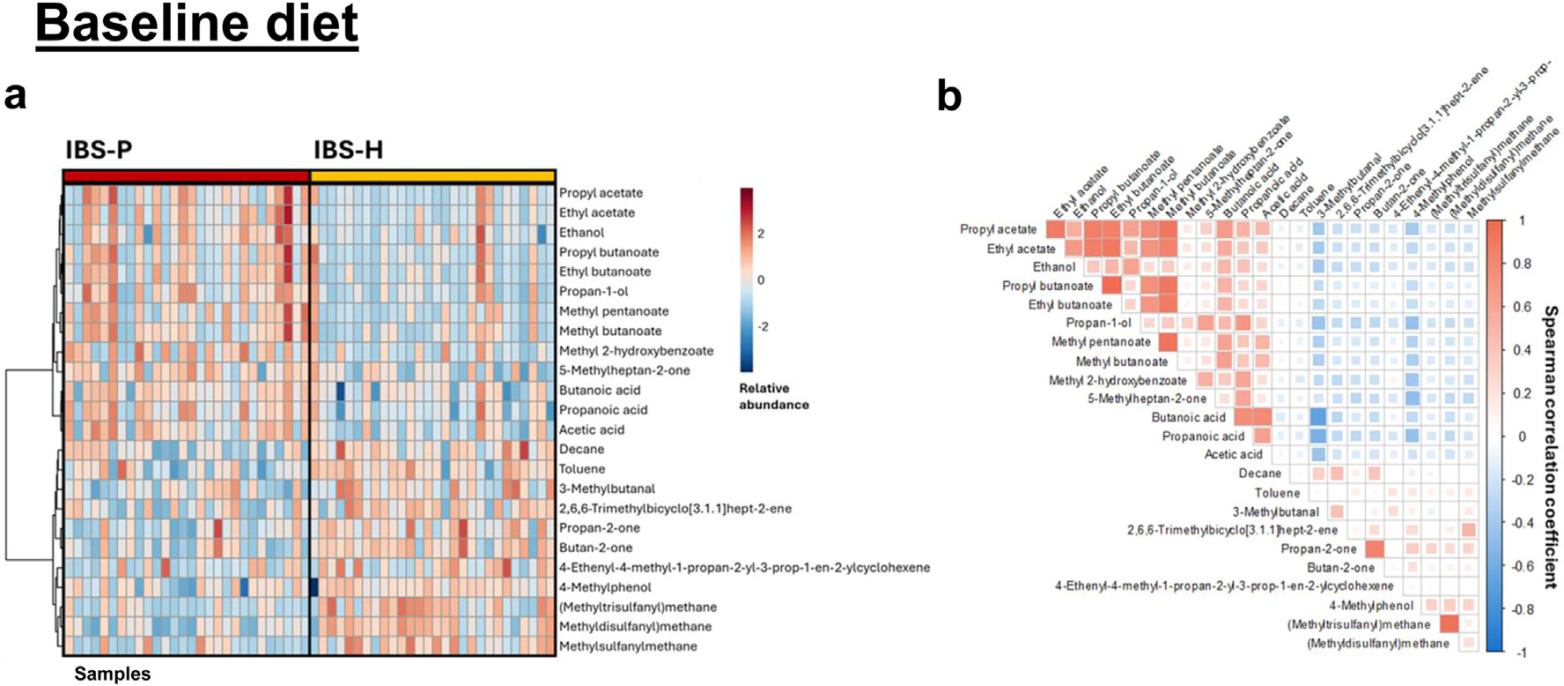
a: Self organising heatmap clustered according to microbial subtype showing the distribution of metabolites during the consumption of the baseline/habitual diet. b: Correlation plot demonstrating relationships between individual volatile organic compounds (VOCs). Positive inter-VOC correlations are observed between the VOCs characterising each metabotype, whereas negative inter-VOC correlations are observed between metabotypes. (N = 56; 28 IBS-P, 28 IBS-H).

A total of 177 VOCs were identified (Supplementary Material 7). At baseline, during the consumption of a habitual diet, the composition of the faecal VOC profiles (both in terms of ‘absolute presence/absence’ using Chi squared [‘absence’ = below limit of GC–MS detection] and ‘relative abundance’ using Wilcoxon Ranked Sum test) were compared across various IBS subgroups (Supplementary Material 8). The use of self-organising metabolite-based heatmaps clustered according to microbial subtype and subsequent correlation plots demonstrated a clear visual pattern of metabolite clustering within the IBS-P and IBS-H comparison (Fig. 2). Such clear discrimination was not observed in any other comparison (other comparisons included: female vs male; IBS-D vs IBS-M; post-infectious IBS vs idiopathic IBS, severe IBS vs non-severe IBS, low FODMAP diet non-responder vs responder, and those who achieved remission [IBS-SSS <75] vs those whose symptoms persisted at some level [IBS-SSS >75]–Supplementary Material 9).

The IBS-P metabotype was characterised by a relative abundance of VOCs associated with carbohydrate fermentation, including SCFAs, SCFA esters, and alcohols (Table 3). The IBS-H metabotype was characterised by a relative abundance of organosulphurs and ketones.

**Table 3:** Summary of the 24 volatile organic compounds demonstrating significant differences in relative abundance at baseline (Unadjusted *p* value determined using Wilcoxon rank test; Adjusted *p* value (q value) determined using according to false discovery rate calculation (FDR); ns, nonsignificant).

Retention time (mins)	Volatile organic compound	Family	Unadjusted *p* value	*q* value (FDR)
**VOCs abundant in IBS-P**				
13.36	Acetic acid	SCFA	<0.05	<0.05
10.10	Propan-1-ol	Primary alcohol	<0.05	<0.05
18.12	Ethyl butanoate	SCFA-ester	<0.05	<0.05
16.89	Propanoic acid	SCFA	<0.05	<0.1
20.13	Butanoic acid	SCFA	<0.05	<0.1
11.08	Ethyl acetate	SCFA-ester	<0.05	<0.1
14.83	Propyl acetate	SCFA-ester	<0.05	<0.1
15.14	Methyl butanoate	SCFA-ester	<0.05	<0.1
7.10	Ethanol	Primary alcohol	<0.05	<0.1
19.14	Methyl pentanoate	SCFA-ester	<0.05	<0.1
21.86	Propyl butanoate	SCFA-ester	<0.05	ns
33.73	Methyl 2-hydroxybenzoate	Benzoate ester	<0.05	ns
**VOCs abundant in IBS-H**				
16.55	(Methyldisulfanyl)methane	Organosulphur	<0.0001	<0.01
25.85	(Methyltrisulfanyl)methane	Organosulphur	<0.001	<0.01
11.09	Butan-2-one	Ketone	<0.001	<0.05
7.97	Methylsulfanylmethane	Organosulphur	<0.001	<0.05
17.02	Toluene	Aromatic hydrocarbon	<0.01	<0.05
7.86	Propan-2-one	Ketone	<0.01	<0.05
25.26	5-Methylheptan-2-one	Ketone	<0.05	<0.1
31.35	4-Methylphenol	Phenol	<0.05	<0.1
24.51	Decane	Alkane	<0.05	<0.1
23.02	2,6,6-Trimethylbicyclo-[3.1.1]hept-2-ene	Terpene	<0.05	<0.1
36.51	4-Ethenyl-4-methyl-1-propan-2-yl-3-prop-1-en-2-ylcyclohexene	Terpene	<0.05	ns
13.10	3-Methylbutanal	Aldehyde	<0.05	ns

Integrated analysis using the mixOmics circosPlot() function revealed that the strongest positive correlations between VOCs and metabolic pathways were between SCFAs/SCFA-esters and pathways for carbohydrate and amino acid metabolism (R = 0.60–0.83) previously identified as enriched in the IBS-P group (Vervier et al., 2022), highlighting the relationships between microbe(s), metabolite(s), and function. A more detailed breakdown of these associations is provided in Supplementary Material 10.

### Controls

**Fig. 3:**
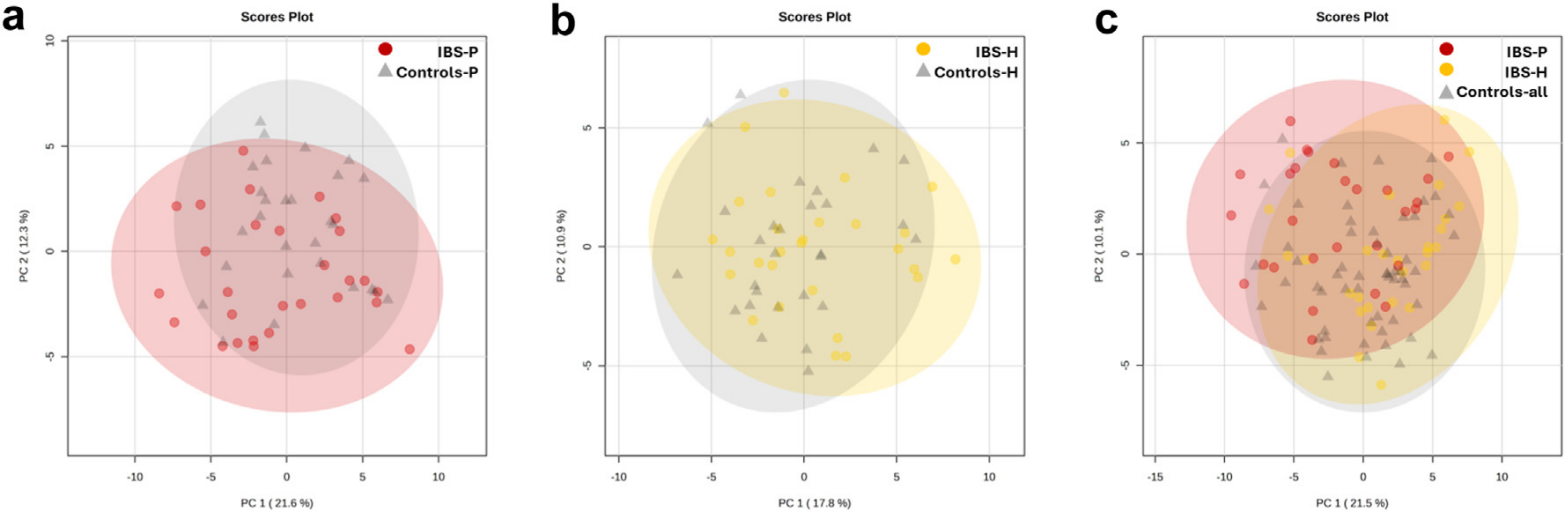
Two-dimensional loading plots following unsupervised principal component analyses demonstrating the degree of similarity/differences between groups. a) IBS-P and their controls; b) IBS-H and their controls; c) IBS-P and IBS-H and all controls (note how the degree of overlap is higher between IBS-H and controls vs IBS-P and controls).

Similarities in the baseline metabotypes were observed between control and their household IBS-case counterparts. Those controls co-habiting with IBS-P closely resembled the VOC profile described in IBS-P cases, and vice-versa in those cohabiting with IBS-H. However, in keeping with the metagenomic data previously described, when considering the control cohort as a single group, this appeared more similar to IBS-H than IBS-P (Fig. 3).

### Baseline class prediction model

**Fig. 4:**
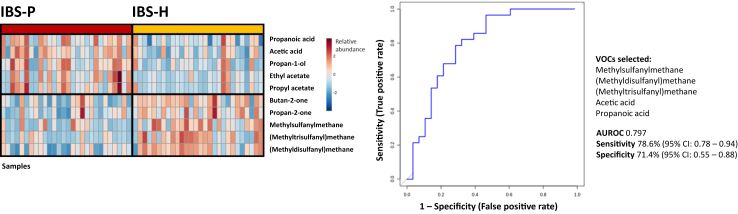
Left: Heatmap demonstrating the key differences between the IBS-H and IBS-P subtypes at baseline. Right: Receiver operator characteristic demonstrating a microbial class (IBS-H/IBS-P) prediction model based on 5 of the most abundant and discriminant volatile organic compounds.

In addition to the pattern observed with respect to the SCFA/SCFA-related VOCs and the specific IBS-P microbial subtype, an inverse VOC pattern emerged (VOCs that were abundant in one group were subsequently low in the other, and vice versa) suggesting that each of the microbial subtypes (IBS-H and IBS-P) could be distinguished based on their specific metabotype. A multivariate biomarker model was constructed using five key VOCs: methylsulfanylmethane, (methyldisulfanyl)methane, (methyltrisulfanyl)methane, acetic acid, and propanoic acid. These VOCs were selected based on patterns observed on visual heatmap inspection and on consideration of their statistical significance following univariate analysis. Using logistic regression and 10-fold cross-validation an area under the ROC curve of 0.797 was achieved to predict group membership, sensitivity 78.6% (95% CI: 0.78%–0.94), specificity 71.4% (95% CI: 0.55–0.88) (Fig. 4).

### Observations moving from ‘baseline’ to ‘four weeks on low-FODMAP diet’

**Fig. 5:**
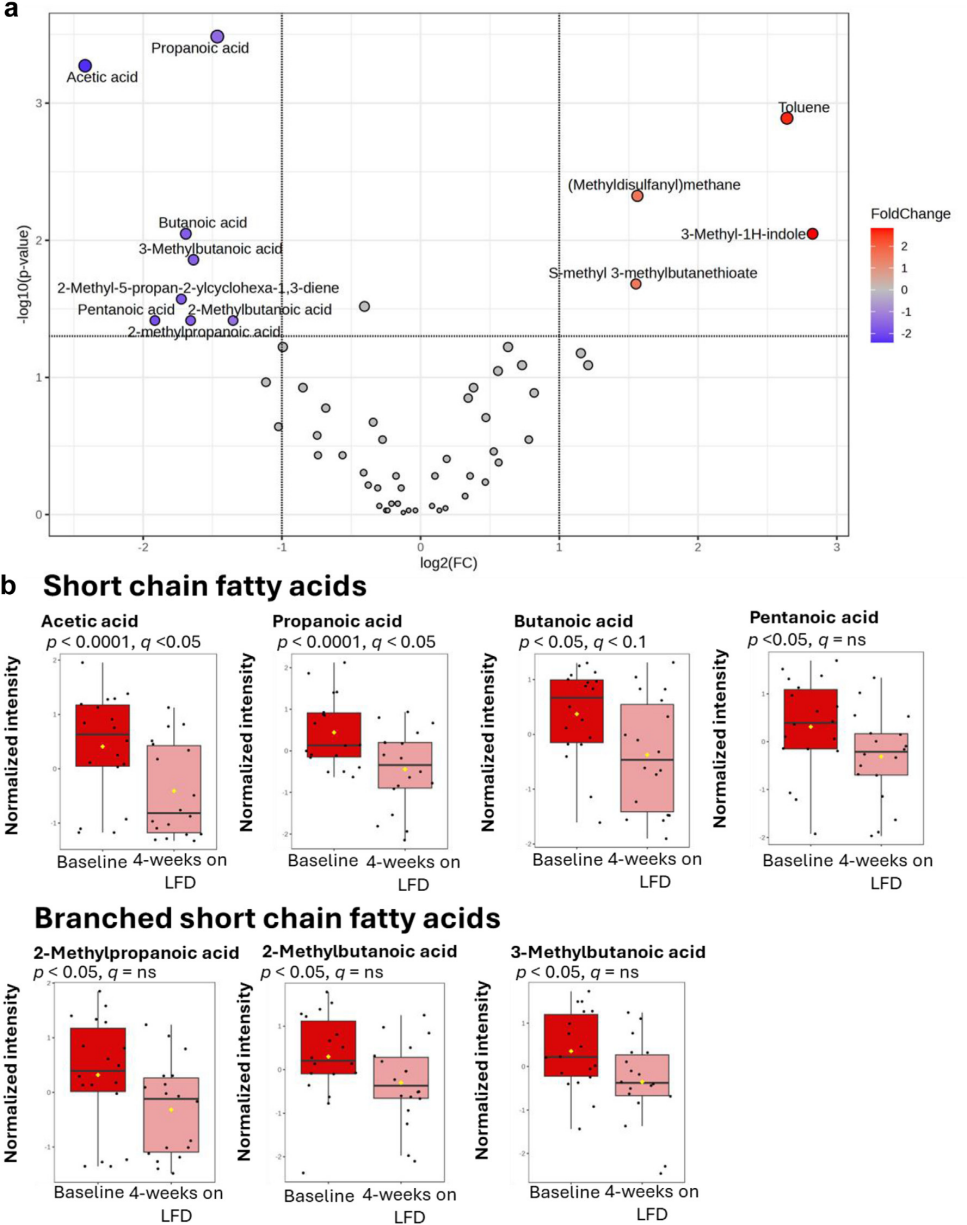
IBS-P group moving from baseline to completion of a 4-week low-FODMAP diet (LFD). N = 18, paired data. a: Volcano plot illustrating the dynamic changes in VOC abundance. Significance parameters set to require—log-2-fold change AND significant unadjusted *p* value (alpha <0.05) on Wilcoxon signed rank test. b: Box plots illustrating the significance of the dynamic changes in SCFA and branched SCFA abundance (Wilcoxon signed rank test).

In the IBS-P group, a significant (>50%) reduction was observed in all seven SCFAs and branched-SCFAs (Fig. 5). The major SCFAs (acetic acid, propanoic acid, butanoic acid) the difference remained significant after adjusting for multiple comparisons (Wilcoxon signed rank test). Within the IBS-H group, no significant differences (Wilcoxon signed rank test) were observed in the relative abundance of any of the SCFAs or branched-SCFAs (Supplementary Material 11) after four weeks of FODMAP exclusion compared to baseline. Only one VOC (4-Methylphenol) demonstrated a significant change within the IBS-H group after the four-week LFD (Supplementary Material 12); this was no longer statistically significant after adjusting for multiple comparisons.

### Observations after completion of a four-week low-FODMAP diet

**Fig. 6:**
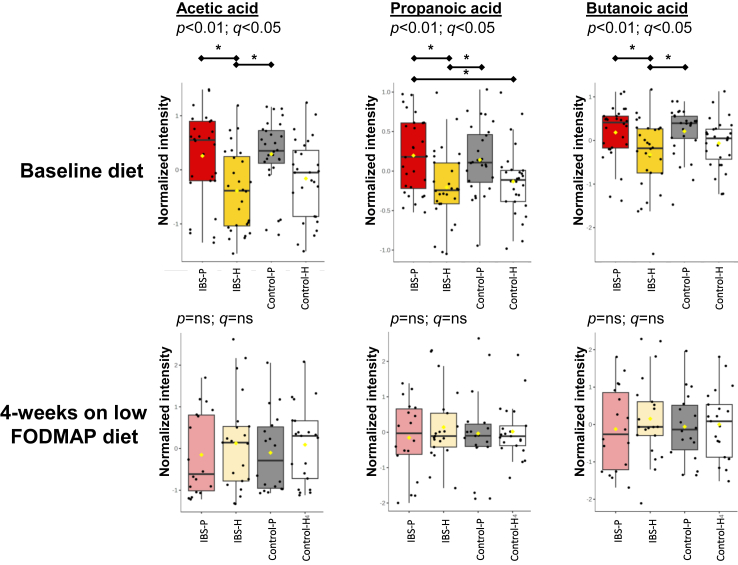
Differences in SCFA abundance at baseline and after completion of a 4-week low-FODMAP diet. Unadjusted *p* values and false discovery rate adjusted q values reported for non-parametric ANOVA. Subsequent significant differences between groups identified on post-hoc analysis are highlighted using the asterisk. N = 78 (39 IBS [18 IBS-P, 21 IBS-H], 39 controls [19 Control-P, 21 Control-H]).

After completion of the four-week low FODMAP diet the relative abundance of SCFA in the IBS-P group (and their paired controls) was reduced to a level comparable to the metabolically adynamic IBS-H group (and their paired controls) (Fig. 6). Groups were compared using Kruskal–Wallis test (Tukey's honest significance test), there were no significant differences in the resultant SCFA abundance between these four groups.

### Observations after FODMAP re-introduction/personalisation at week 16

High study attrition rates between visit B and visit C, particularly within the IBS-P group (61.1% attrition IBS-P; 33.3% attrition IBS-H), compromise the robustness of any observations made at this point. FODMAP scores returned to baseline levels in each group (median 7.5 [range 3–10]). However, this was not accompanied by metabolic instability within either metabotypes. No VOCs in the IBS-H group demonstrated a significant change, and just one VOC (6,6-dimethyl-2-methylidenebicyclo [3.1.1]heptane) in the IBS-P group demonstrated a significant positive fold change—the biological significance of this is uncertain. No statistically significant changes in symptom severity scores were observed (paired samples t-test).

## Discussion

We have defined two distinct measurable metabotypes corresponding with the microbial subtypes described previously by Vervier et al. Where the ‘dysbiotic’ microbiome of those with the IBS-P subtype was ‘corrected’ by the low FODMAP diet (which after four-weeks resembled that of the healthy group), an equally dynamic change in the IBS-P metabotype has followed, providing a metabolic hypothesis for symptom generation and the enhanced response to FODMAP restriction observed in this group.

The human small intestine is ill-equipped to enzymatically degrade FODMAPs, along with the majority of ingested dietary fibre. Instead, such substrates are delivered to the colon unaltered where fermentation by the host colonic microbiota occurs.^25^ Whilst the production of gas and osmotically active by-products is thought to drive symptoms,^26^ the contribution from microbial derived neuro-active metabolites is increasingly recognised in IBS.^27^^,^^28^ Indeed, Tuck et al. recently demonstrated the pronociceptive potential of faecal supernatants from patients responsive to the low FODMAP diet when transplanted into mice, which was lost after FODMAP restriction.^27^

A principal feature of the baseline IBS-P microbiome was a relative abundance of diverse Firmicutes species enriched in genes for carbohydrate (and amino acid) metabolism,^12^ suggesting that people in this group are primed to generate SCFAs (potentially in surplus amounts) if exposed to FODMAPs. Restricting FODMAPs in those with a baseline microbiome primed toward excessive fermentation seemingly starves the resident pathobionts of substrate. Not only does this result in a net reduction in fermentative biomass, but it also reduces the abundance of SCFAs (and other key metabolites). We have now demonstrated strong positive correlations between bacterial metabolic pathways and SCFAs/SCFA-esters using integrated analysis, linking bacterial mechanism to functional metabolites. The observation that the composition of the IBS-P metabolome shifted toward that seen in those with the ‘healthy’ microbiota, and that this was accompanied by a significant reduction in IBS symptoms and abdominal pain, supports the notion that the IBS metabolome is a key modifiable contributor to the pathophysiology of IBS and visceral hypersensitivity.^12^

A key finding of our study is the extension of the two identified metabotypes beyond the individuals with IBS to include their healthy household counterparts. This observation likely reflects a degree of microbial crosstalk and shared dietary habits within households. Crucially, despite this apparent sharing of metabotype, IBS symptoms are not shared with the controls. These findings add another layer of complexity to our understanding of IBS, suggesting that whilst a specific microbial subtype may indeed contribute to the formation of a distinct metabotype, the generation of IBS symptoms must depend on other neurobiological factors specific to those with IBS.

SCFAs are essential for gut health, and deficiency (both in faeces and in plasma) is increasingly recognised in the pathogenesis of various disease states.^29^, ^30^, ^31^, ^32^ Whilst the relevance of plasma SCFA concentrations in IBS remains uncertain, evidence continues to emerge implicating the potential role of faecal SCFAs in the pathogenesis of IBS, such that a SCFA-gut-brain axis has been proposed in the recent literature.^19^ Indeed, SCFAs comprise a significant proportion of the metabolites produced as a result of microbial fermentation/metabolism, and depending on the niche within which they are produced, these metabolites have been shown to harbour pro-nociceptive potential.^19^^,^^33^

Consistent with a pro-nociceptive effect, SCFAs have been shown to stimulate serotonin release from enterochromaffin cells,^34^ stimulate sensory nerves innervating the bowel, and promote visceral hypersensitivity following colorectal butyrate enema.^33^^,^^35^ Further, 3-methylbutanoic acid (better known as isovaleric acid) has been shown to be both pro-nociceptive and modifiable in individuals with visceral hypersensitivity, activating enterochromaffin cells via G-protein coupled receptors and resulting in the increased sensitivity of primary afferents via a serotonin dependent mechanism.^18^^,^^36^ More recently, the findings from Barki et al. demonstrate a process whereby an abundance of microbially derived propanoic acid can activate sensory neurons via interaction with their cognate free fatty acid receptors (FFA2 and FFA3).^19^ The authors demonstrated the potential to manipulate this SCFA-gut-brain-axis using synthetic agonists. Whilst this mechanism is not yet fully understood, the biological plausibility of these findings and the potential for the manipulation of these pathways represents an exciting therapeutic target in IBS.

Whilst understanding of the potential algesic effects of SCFAs continues to evolve, a link between SCFAs and transit has been consistently demonstrated. However, whilst SCFAs are associated with faster transit,^37^ whether their measurement in faeces simply represents the rapid propulsion and excretion of proximal colonic content, or whether the SCFA metabolites themselves contribute to the physiology of rapid transit is less clear. Recent data from a murine study demonstrate that transit time was reduced following the colonic infusion of 1% acetic acid, supporting the role of SCFA imbalance in the pathogenesis of non-constipated IBS.^38^ Further, a significant positive correlation was identified between propanoic acid concentration and rapid colonic transit in an exclusive IBS-D cohort.^39^ As one might expect, faster colonic transit times correlate with increased stool frequency and more-liquid stool form; however in IBS faster transit does not reliably associate with global symptom severity or pain,^40^ supporting the notion that it is the microbially derived gut metabolites that are mediating these symptoms.

Perhaps least understood, but most intriguing, are the potential mechanisms by which SCFAs influence neuropsychiatric conditions and psychological functioning.^41^ In addition to the relationship between SCFA and the biosynthesis/release of centrally acting neurotransmitters such as serotonin,^34^ SCFAs (particularly propanoic- and butanoic acid) are capable of inhibiting the activity of intracellular histone deacetylases (HDACs)—a group of enzymes involved in both cognitive development and neuropsychiatric disease.^41^^,^^42^ Though the bi-directional relationship between IBS and psychological disease is increasingly recognised,^43^ recent expert opinion suggests that it is premature to conclude whether SCFAs play a favourable or unfavourable role in their development. Moreover, it has been acknowledged that SCFAs might play an indirect role in the development of psychological disorders as a result of the impact of worsening physical symptoms.^41^

It has been previously suggested that restricting dietary fermentable carbohydrate substrate risks the depletion of the SCFA pool in IBS,^44^, ^45^, ^46^ leading to concerns that long-term FODMAP restriction may have deleterious effects on the gut microenvironment. The strict long-term restriction of FODMAP restriction has therefore been cautioned by some.^45^^,^^47^ Our findings provide an alternative perspective on this hypothesized risk, illustrating that the SCFA profile was not depleted by the low FODMAP diet, but rather it recalibrated toward the levels observed both in healthy non-IBS controls and those with the IBS-H microbial subtype. This unique finding suggests that those with IBS-P group might be better considered as ‘fermenters’ in whom there exists an allowance for the manipulation of SCFA metabolites, and that this reduction might be favourable (or indeed, therapeutic) rather than pathological. Of course, further longitudinal studies are now required.

We are not the first to identify a sub-group of IBS patients enriched in SCFAs. Indeed, in the last decade a handful of studies have linked this emerging metabotype either with an increased overall response rate,^48^ or higher magnitude responses to FODMAP restriction (and similar dietary interventions).^49^, ^50^, ^51^ Chumpitazi et al. in their trial of FODMAP restriction in children with IBS concluded that the identification of a microbial profile with greater saccharolytic capacity may serve as a biomarker for higher magnitude responders^51^; our findings have advanced this hypothesis further still. However, not all findings align with ours.

So et al.,^52^ in their recent systematic review found no differences in total (or specific) faecal SCFA concentration between those completing a low FODMAP diet versus control diets. Given that we have now demonstrated a cohort of individuals (comprising both IBS and healthy controls) in whom FODMAP restriction made no significant microbial/metabolic impact, we would speculate that findings might have differed if IBS patients were subclassified by microbial/metabolic subtype. We further acknowledge that a bacterial profile is yet to emerge with certainty across geographically discrete IBS populations. For example, whilst Bennet et al.^53^ did indeed demonstrate that responders and non-responders to the low FODMAP diet had different microbiota profiles at baseline, it was the *non-responders* that were characterised surplus of Firmicutes species and saccharolytic bacteria. This highlights the requirement for further, well-designed, high-powered studies encompassing a multi-omics approach to further address this intriguing biological gap in understanding.

Our study does have limitations. As with the index work from Vervier et al., the sample size was relatively modest and confined geographically to Cambridge and the surrounding areas. Furthermore, the absence of precise measurements for dietary fibre consumption limits our ability to assert with confidence that the differences in SCFA abundance between metabotypes are driven entirely by the microbiome. The lack of a sham dietary group limits the strength of the clinical response findings, and the lack of transit time assessment means that questions pertaining to transit, symptoms and SCFA abundance remain unanswered. Our analysis was post-hoc, and a retrospective power calculation suggests that a sample size of >400 is required to provide 80% power at 10% significance (using FDR adjustment) to validate our findings. Another limitation of our study is the large number of metabolites analysed relative to the sample size, and although measures have been taken to mitigate for this, we acknowledge that this may introduce challenges in generalizability and potential overfitting. Coupling these with the paucity of literature pertaining to this emerging IBS subgroup, replication is required.

Maintaining complete experimental oversight is a well-documented challenge in studies involving a dietary intervention, and by the very nature of the study we acknowledge that there are potential risks of referral bias at recruitment and the Hawthorne effect during intervention. Also, the IBS-P group had a tendency toward a less healthy lifestyle at baseline (more smokers, higher BMI, marginally higher alcohol intake)—and whilst these differences were not statistically significant, we acknowledge that they could have contributed to differences in the baseline gut microbiota.^54^, ^55^, ^56^ Furthermore, whilst psychological status is recognised as a key factor when evaluating response to IBS therapies (particularly diet), it was unmeasured in this study. Whether dietary intervention led to more noticeable improvements in general well-being and psychological status in either group is uncertain, however we acknowledge the possibility that the IBS-P group (who were generally ‘less healthy’ at baseline) potentially stood to gain more from dietetic engagement and dietary intervention.

From an analytics perspective, the semi-quantitative nature of our GC–MS analysis limits the precision with which the absolute abundance of the detected VOCs can be determined. Future studies should focus on expanding our findings by further defining these metabotypes using relative quantification. Finally, at the time of analysis, the lifespan of some of the samples exceeded 6-years, although samples were strictly maintained in −80 °C storage conditions throughout.

We have found a distinct fermentative and metabolically dynamic metabotype, in our cohort of non-constipated IBS patients, which is characterised by SCFAs and associates with the previously identified IBS-P microbial subtype. Whilst this metabotype extends beyond IBS and into controls, those with IBS are characterised by greater symptom severity at baseline and an enhanced clinical response to the low FODMAP diet than those without this metabotype. A model consisting of key metabolites enabled the prediction of microbial subtype with accuracy, the identification of which could better justify the restriction of FODMAPs whilst vindicating the trade-off between meaningful symptom-improvement and the challenges associated with this burdensome therapeutic approach. Additionally, these results provide reassurance that the pragmatic restriction of FODMAPs may not lead to SCFA depletion, but instead to SCFA recalibration. It is noteworthy that in the small number of participants who completed the FODMAP reintroduction phase, the metabolomic changes appeared less pronounced compared to the initial restrictive phase, suggesting a potentially less drastic impact during reintroduction. Whether this reflects an enduring effect on the microbiome, or a preference to avoid certain fermentable foods, after the re-introduction phase will require further research.

## Contributors

TEC, MP, and CP planned the study. SM, JdlRN and MP conducted the initial clinical study including patient assessments and recruitment, data collection, and stool collection. TEC, RS, UZI and CP contributed to the analysis of the data and reported results. TEC, RS, and UZI generated figures and tables. TEC, RS, SM, DCB, DMP, MP, and CP contributed to scientific discussions. TEC, SM, DCB, MP, and CP drafted the manuscript. TEC, RS, SM, DCB, JdlRN, UZI, DMP, MP and CP contributed to the revision of the manuscript. TEC and CP verified the data and are responsible for the overall content as guarantors. All authors read and approved the final version of the manuscript.

## Data sharing statement

All raw metabolomic data is available on the Mendeley Data platform: https://doi.org/10.17632/5cw5rghg7m.1. Raw metagenomic sequencing data are accessible under ENA Study Accession Number: ERP021923, and study descriptive metadata are available at: http://github.com/kevinVervier/IBS.

## Declaration of interests

DCB has received grants from AstraZeneca, Biotechnology and Biological Sciences Research Council, Crohn's and Colitis Foundation, GlaxoSmithKline and Metrion. MP has received research/educational grants and/or speaker/consultation fees from Helmsley Trust, AstraZeneca, Pfizer, Galapagos and Gilead research grants. TEC, RS, SM, JdlRN, UZI, DMP, and CP have no relevant competing interests to declare.

## Appendix A Supplementary data

Supplementary Materials

Caption for Supplementary Materials
