# Supplementary material for: Microbiome-driven IBS metabotypes influence response to the low FODMAP diet: insights from the faecal volatome
Source: eBioMedicine. 2024 Aug 22;107:105282. doi: 10.1016/j.ebiom.2024.105282 (PMC11388012; doi:10.1016/j.ebiom.2024.105282)

**Supplementary Material 1:**

Severity metrics used in study.

| **Irritable Bowel Syndrome Symptom Severity Score (IBS-SSS)** | | |
| --- | --- | --- |
| **Parameter (scored on visual analogue scale from 0-100)** | **Total score**  **(/500)** | **Pain composite score (/200)** |
| Severity of abdominal pain | 0 – 100 | 0-100 |
| Number of days that you get pain in every 10 days (number multiplied by 10) | 0 – 100 | 0-100 |
| Severity of abdominal distension/tightness | 0 – 100 | - |
| Satisfaction with bowel habit | 0 – 100 | - |
| How much IBS interferes with life in general (0% = not at all; 100% = completely) | 0 – 100 | - |
| **Classification according to symptom severity** | | |
| Remission | <75 | - |
| Mild | 75-174 | - |
| Moderate | 175-299 | - |
| Severe | 300-500 | - |
| **Response definition** | | |
| 50-point reduction in total IBS-SSS score | - | - |

FODMAPs were grouped into 7 categories (lactose, fructose, fructans from fruit and vegetables, fructans from cereal, fructans from garlic and onion, galactoligosaccharides and polyols). Ingestion was scored from 0-2 according to intake (assessed by way of 7-day food diary).

A low intake was scored 0, moderate intake 1 and a high intake 2. Possible scores ranged from 0-14.

|  | **FODMAP ingestion score** | | |
| --- | --- | --- | --- |
| **FODMAP category** | Low (0) | Moderate (1) | High (2) |
| Lactose |  |  |  |
| Fructose |  |  |  |
| Fructans from fruit and vegetables |  |  |  |
| Fructans from cereal |  |  |  |
| Fructans from garlic and onion |  |  |  |
| Galactoligosaccharides |  |  |  |
| Polyols |  |  |  |
| **Total score: --/14** | | | |

**Supplementary Material 2:**

Experimental samples were pre-incubated at 60^o^C for 20 minutes before exposing the solid phase microextraction fibre to the faecal headspace for 30 minutes. The carrier gas used was helium (99.996% purity, BOC, Sheffield, UK), flow rate was set at 1 ml/min. The total GC-MS run-time was 42 minutes – consisting of an initial 2-minute hold at 40^o^C, followed by a sequential 5^o^C-per-minute ramp peaking at 220^o^C where it was held for 4-minutes.


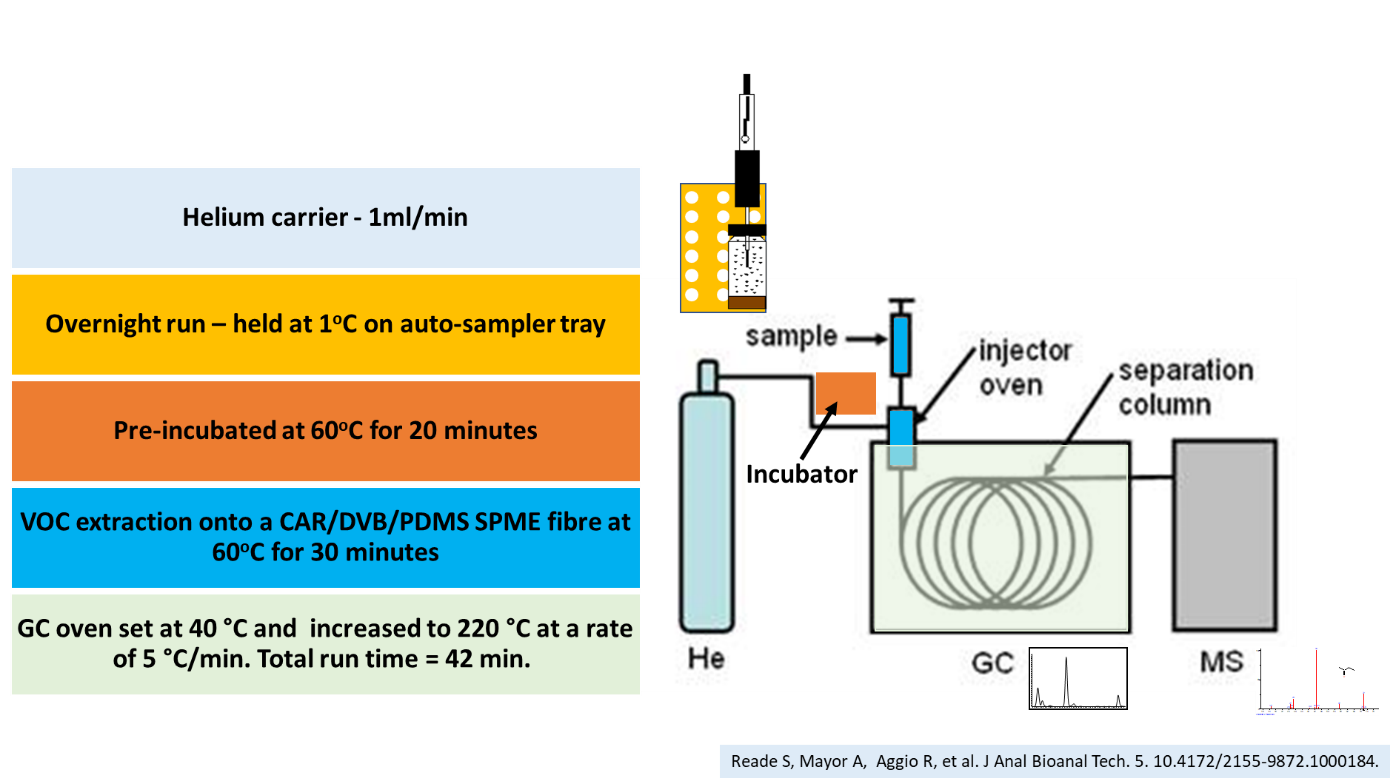


**Supplemental Material 3:**

Depiction of a typical SPME-GC-MS ‘run’ – demonstrating how samples were racked onto the Combi-PAL autosampler tray, and how quality control samples were built into the run-method. A total of 6 compounds were excluded as contaminants from the final analysis, the other 6 were tentatively included having been identified in several other faecal VOC studies (Supplementary Materials 7).

**
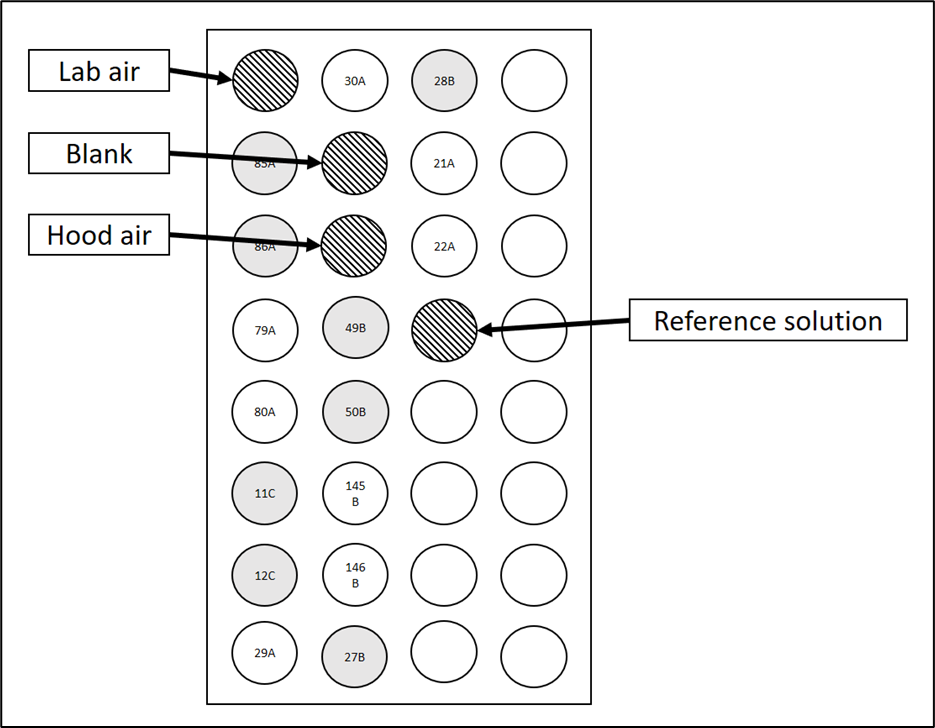
**

| **Quality control sample** | **Attributes** | **Rationale** |
| --- | --- | --- |
| **Lab Air** | - Uncapped empty vial in which the SPME fibre would sit and sample the air for 20 minutes. - First sample on every batch. | Analysed to identify, and potentially exclude, possible environmental contaminants from the analysis. |
| **Blank** | - Capped empty 10ml headspace vials. - Run after every 8 samples. | Analysed to reduce the risk of VOC carry-over on the SPME fibre between samples. |
| **Reference solution** | - Solution made in lab – mixed to 1:10,000 solution. - Three compounds selected with known retention time (RT) and mass-to-charge (m/z) ionic profiles:   - Pyridine (RT 16.91min)   - Gamma-Valerolactone (5-methyloxolan-2-one) (RT 27.50min)   - N-Methyl-2-Piperidone (RT 33.97min). - Final sample to be run on every batch. | This QC standard processed under the same experimental conditions as the faecal samples, ensured that SPME-GC-MS performance was measurable and consistent. |
| **Hood air** | - One ‘hood air’ sample added per ‘run’, until all analysed (n=9). | Representative of the ‘hood’ (class II MSC) working environment to which the samples were exposed during aliquoting. Analysed to identify, and potentially exclude, possible environmental contaminants picked up during exposure to the working environment within the class-II MSC. |

**Supplemental Material 4:**

Metabolite Inclusion criteria.

| **NIST nomenclature** | **Max score** | **Minimum score for inclusion** | **Definition** |
| --- | --- | --- | --- |
| Direct match | 999 | 800 | Comparison of the candidate compound’s spectral peaks to those of the peaks in the library’s spectra. |
| Reverse match | 999 | 800 | The match factor when the peaks in the candidate compound’s spectrum that are not in the library’s known reference spectrum are ignored, aiding the identification of compounds when >1 compound is co-eluting within the chromatogram. |
| Probability (%) | 100 | 60 | The relative probability that the candidate compound is represented by the compound within the NIST library that it has been matched with. |

**Supplemental Material 5:**

mixOmics model parameters

Prior to input of data into the DIABLO model, Metacyc pathway data were filtering to 0.1% to remove sparse features. The pathway data were then normalised by Total Sum Scaling normalisation (TSS) followed by centred log-ratio (CLR). VOC data were processed and normalised as described in the main methods. The balanced error rate (BER) was determined using the perf() function and a 10-fold cross validation. The BER was maintained below 20% for the first 3 components using the centroids distance, which were then selected to be included in the model. The tune.block.splsda function was used to choose the optimum number of variables for each block (VOCs and pathways). Using these optimal variables, the final DIABLO model was constructed. The circosPlot() function was used to generate correlation coefficients and visualise correlations between blocks (as shown in **Supplemental Material 10**).

**Supplemental Material 6:**


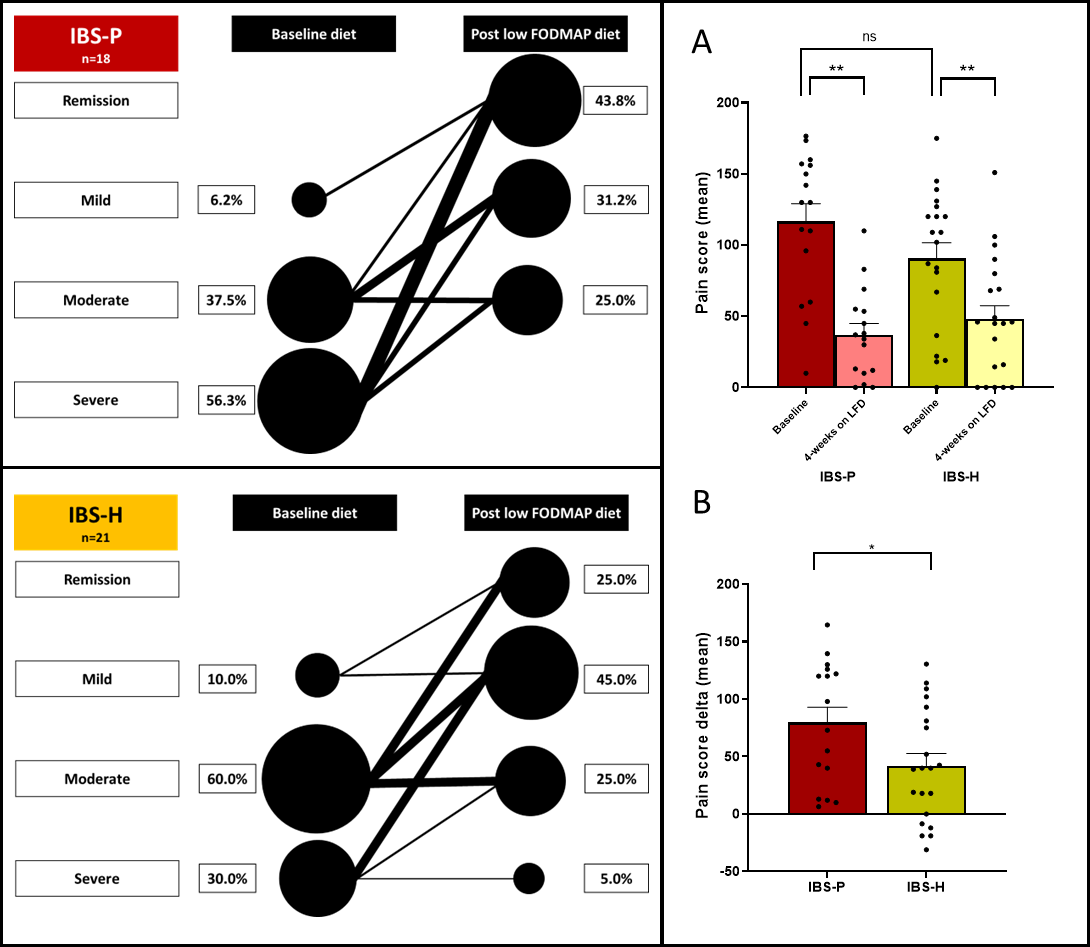


**Supplemental Material 7:**

Complete library of volatile organic compounds included in analysis (n=177)

**Supplementary Material 8**

| **Comparison** | **Number of VOCs demonstrating a significant difference at baseline** | |
| --- | --- | --- |
|  | **Absolute presence/absence** | **Relative abundance** |
| **Controls vs IBS** | 6 | 0 |
| **Female vs Male (IBS)** | 0 | 0 |
| **IBS-D vs IBS-M** | 4 | 0 |
| **IBS-P vs IBS-H** | **22** | **20** |
| **Idiopathic vs PI-IBS** | 4 | 1 |
| **Non-responder vs Responder** | 0 | 0 |
| **Non-severe vs Severe** | 0 | 0 |
| **‘Persister’ vs ‘Remitter’*** | 3 | 0 |

**
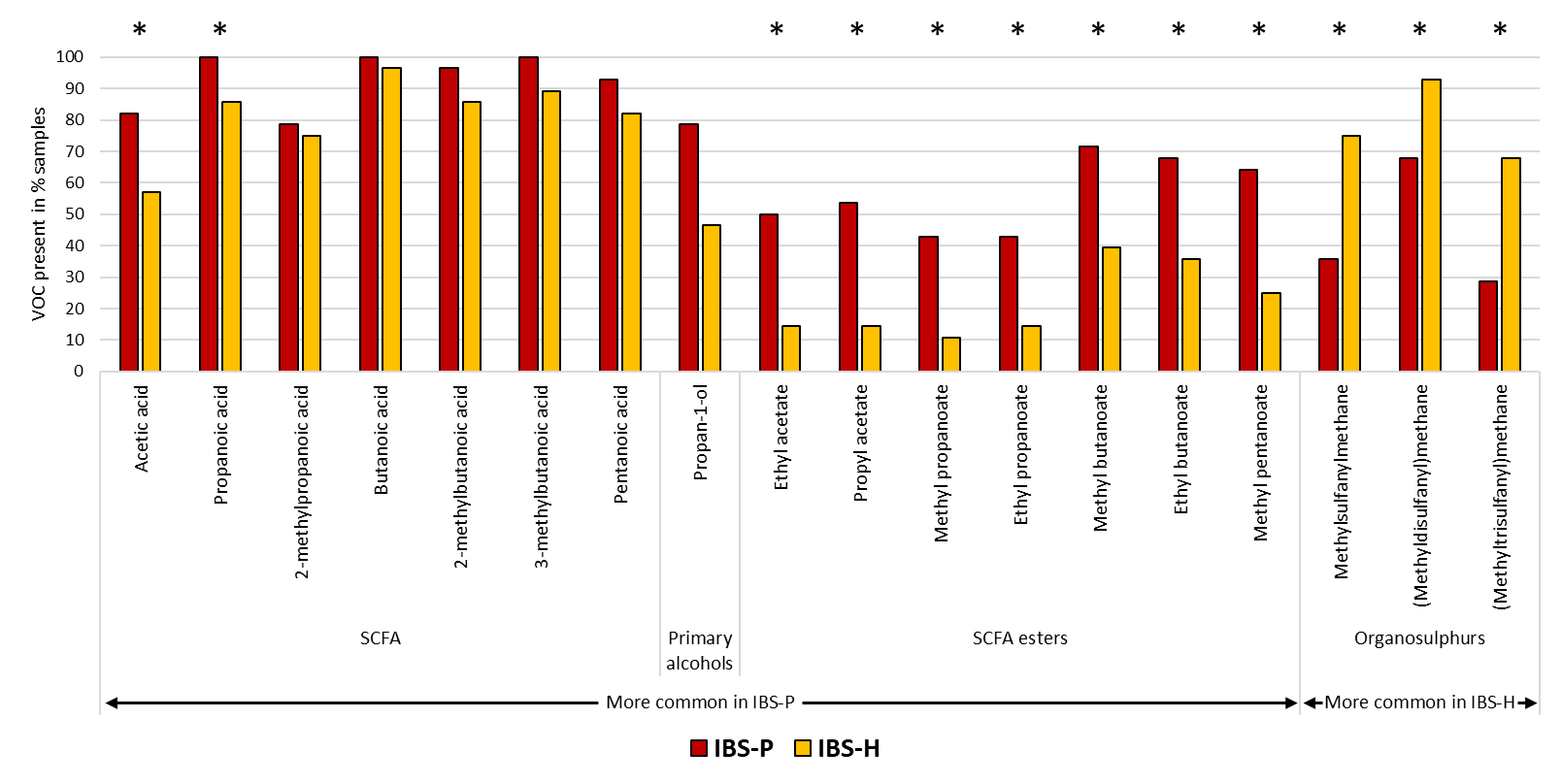
**

**Supplementary Material 9:**


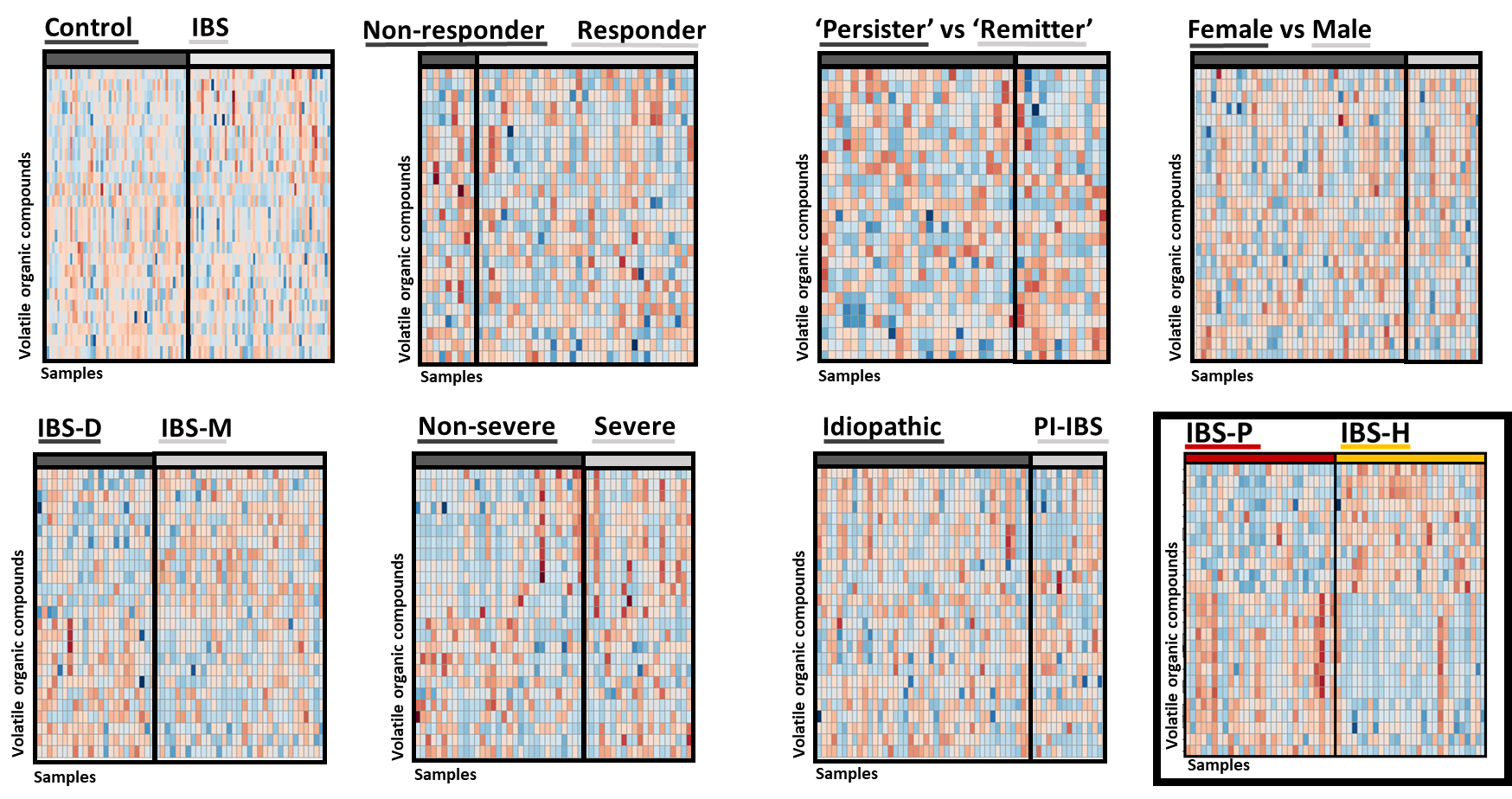


**Supplementary Material 10:**

Circos Plot demonstrating strong correlation between SCFAs and closely associated metabolites with several metabolic pathways. Limited to correlation cut-off of 0.75.


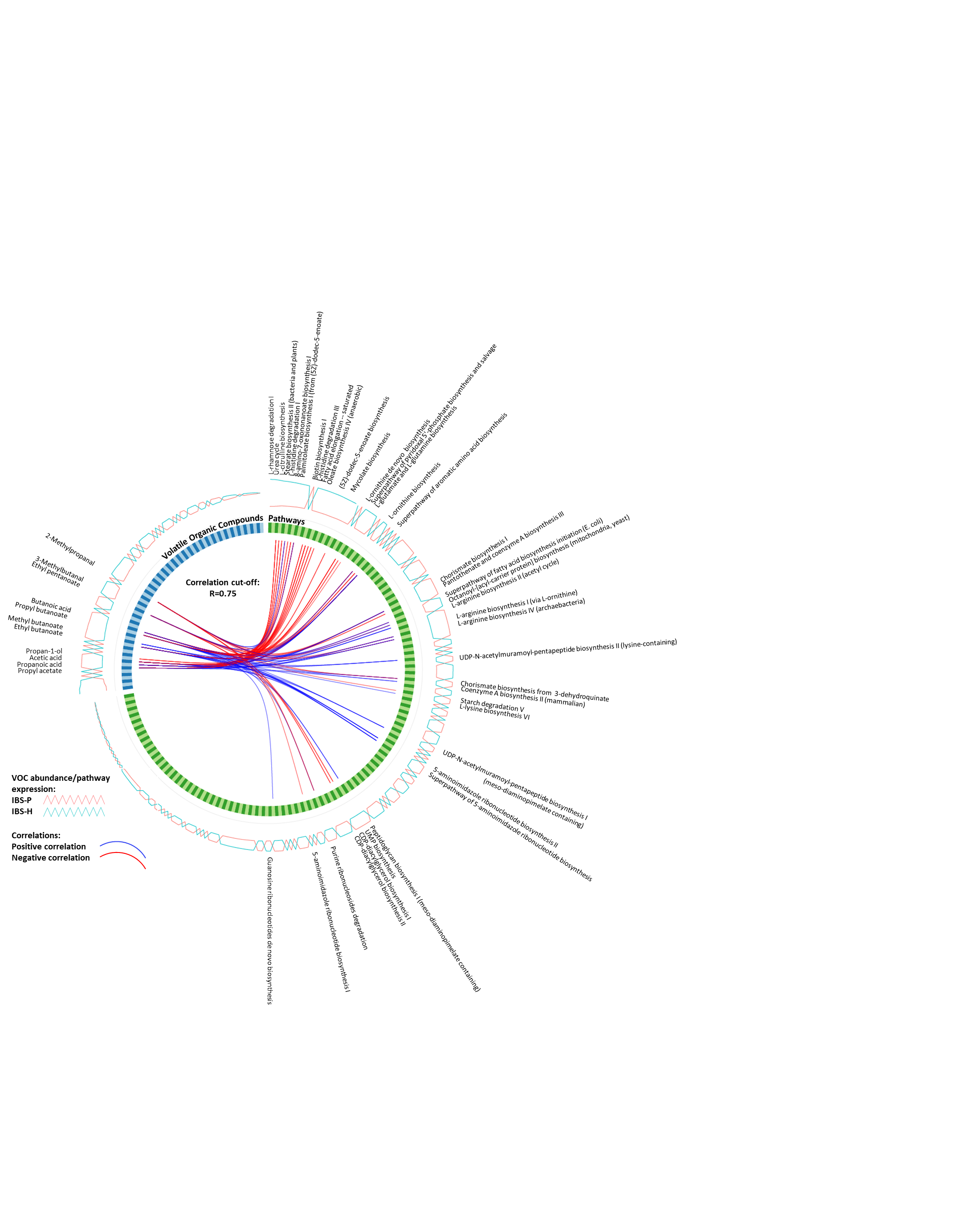


**Supplementary Material 11:**

Box and whisker plots illustrating the absence of significant changes in the relative abundance of SCFA or BSCFA metabolites in the IBS-H group.


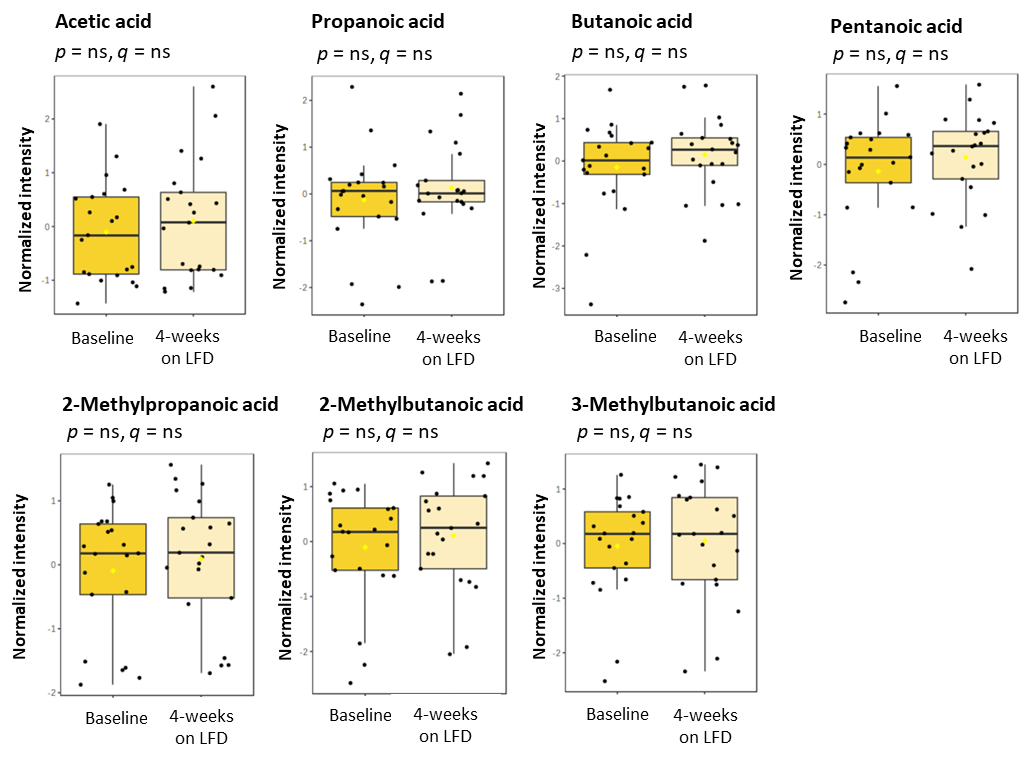


**Supplementary Material 12**

Volcano plot highlighting the lack of dynamic change in VOC abundance in the IBS-H group when moving from baseline diet to completion of a four-week low FODMAP diet. Only one VOC (4-methylphenol) demonstrated both a log2 fold-change and a significant unadjusted p value after univariate analysis by way of Wilcoxon signed rank test.


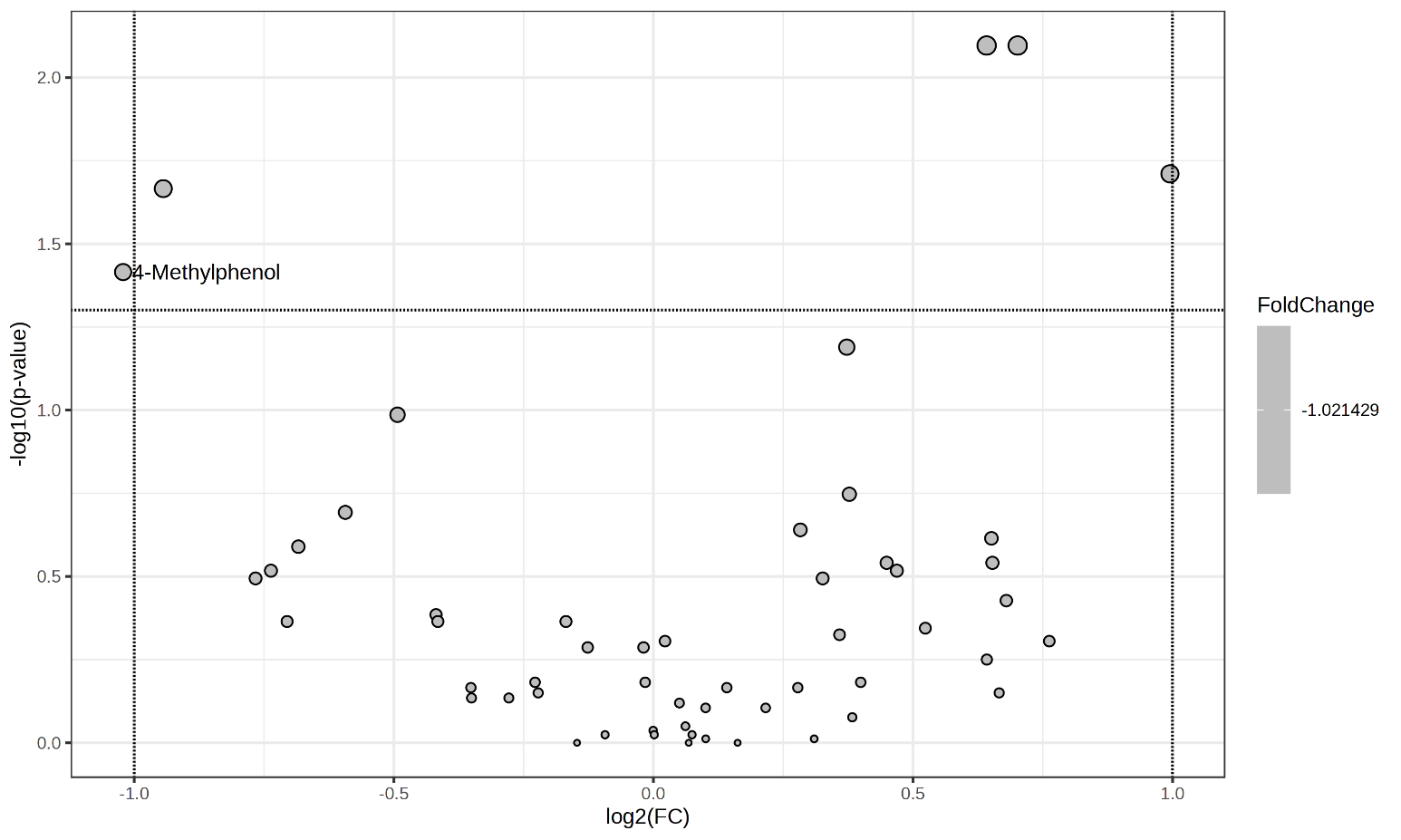

Supplement: Supplementary Materials [file mmc1.docx]
