# Supplementary material for: Microbiome-driven IBS metabotypes influence response to the low FODMAP diet: insights from the faecal volatome
Source: eBioMedicine. 2024 Aug 22;107:105282. doi: 10.1016/j.ebiom.2024.105282 (PMC11388012; doi:10.1016/j.ebiom.2024.105282)
Supplement: Caption for Supplementary Materials [file mmc2.docx]

**Supplementary Material 1**

**IBS-SSS table caption:**

*IBS symptom severity score: Francis CY, Morris J, Whorwell PJ. The irritable bowel severity scoring system: a simple method of monitoring irritable bowel syndrome and its progress. Aliment Pharmacol Ther. 1997 Apr;11(2):395-402. doi: 10.1046/j.1365-2036.1997.*

**FODMAP score table caption:**

*FODMAP score: McIntosh K, Reed DE, Schneider T, et al. FODMAPs alter symptoms and the metabolome of patients with IBS: a randomised controlled trial. Gut. 2017 Jul;66(7):1241-1251. doi: 10.1136/gutjnl-2015-311339.*

**Supplementary Material 2**

**Figure caption:**

*Modified solid phase microextraction-GC-MS method utilised.*

**Supplementary Material 3**

**Figure caption:**

*Example SPME-GC-MS ‘run’.*

**Table caption:**

*Summary of quality control samples (GC-MS – gas chromatography mass spectrometry; Quality control sample summary. MSC – medical safety cabinet; RT – retention time (minutes); SPME – solid-phase micro-extraction; QC – quality control; VOC – volatile organic compound).*

**Supplementary Material 4**

**Table caption:**

*Inclusion criteria required for candidate metabolites identified by GC-MS (gas-chromatography mass-spectrometry).*

**Supplementary Material 5**

NA

**Supplementary Material 6**

**Figure caption:**

*Network analysis demonstrating disease severity dynamics following low FODMAP diet according to microbial subtype. Left: Network analysis illustrating the flux in IBS symptom severity after completion of a 4-week low FODMAP diet. Line thickness correlates with the percentage of patients following that vector from Baseline to Post low FODMAP diet. Symptom severity is scored according to the Irritable Bowel Syndrome Severity Scoring System (IBS-SSS). Right: Bar charts illustrating the differences in pain scores (error bars depict standard error of the mean); A – Comparison of pain scores on baseline diet and after 4-weeks on low-FODMAP diet; B – Direct comparison of pain score deltas (* p<0.05; **p<0.01; ns – nonsignificant [paired and independent t-tests]).*

**Supplementary Material 7**

**Table caption:**

*Complete library of volatile organic compounds included in analysis, n=177 (**Tentative compound assignments: Compound identified in control samples and therefore has potential to be a contaminant compound [Supplementary Material 4]).*

**Supplementary Material 8**

**Table caption:**

*Differences in metabolic profiles when comparing IBS subgroups. Absolute presence/absence assessed by Chi Squared test (p<0.05). Relative abundance assessed using Wilcoxon rank (p<0.05). [*’Remitter’ describes a patient who entered remission according to the Irritable Bowel Syndrome Severity Scoring System (IBS-SSS <75); ‘Persister’ describes a patient who continues to experience symptoms (IBS-SSS>75].*

**Figure caption:**

*Differences in the absolute presence/absence of volatile organic compounds at baseline when comparing IBS-H and IBS-P. Significance assessed using Chi Squared test); * p<0.05.*

**Supplementary Material 9:**

**Figure caption:**

*Series of heatmaps depicting the differences in the distribution of volatile organic compound between subgroups (Female/male status determined according to biological sex; IBS-D – diarrhoeal subtype; IBS-H – ‘healthy microbiota subtype’; IBS-M – mixed subtype; IBS-P – ‘pathological microbiota subtype’; PI-IBS – post-infectious IBS; ‘Persisters’ – individuals who persisted with IBS-SSS scores >75; ‘Remitters’ – individuals who achieved remission-range IBS-SSS scores [<75/500]).*

**Supplementary Material 10:**

**Figure caption:**

*Circos plot showing correlation between SCFAs and SCFA esters and the expression of specific metabolic pathways.*

**Supplementary Material 11:**

**Figure caption:**

*Changes in the relative abundance of SCFAs in the IBS-H metabotype when moving from baseline diet to low-FODMAP diet (ns = non-significant [Wilcoxon signed rank test]).*

**Supplementary Material 12:**

**Figure caption:**

*Volcano plot highlighting the lack of dynamic change in VOC abundance in the IBS-H group when moving from baseline diet to completion of a four-week low FODMAP diet.*
